# *Candidatus* Liberibacter americanus induces significant reprogramming of the transcriptome of the susceptible citrus genotype

**DOI:** 10.1186/1471-2164-14-247

**Published:** 2013-04-12

**Authors:** Valéria Mafra, Polyana K Martins, Carolina S Francisco, Marcelo Ribeiro-Alves, Juliana Freitas-Astúa, Marcos A Machado

**Affiliations:** 1 Centro de Citricultura Sylvio Moreira, Instituto Agronômico de Campinas, Cordeirópolis, São Paulo, Brazil; 2 Universidade Estadual de Campinas, Campinas, São Paulo, Brazil; 3 Embrapa Cassava & Fruits, Cruz das Almas, Bahia, Brazil; 4 Laboratório de Pesquisa em Farmacogenética / Instituto de Pesquisa Clínica Evandro Chagas (IPEC) – Fiocruz, Rio de Janeiro, Brazil

**Keywords:** Gene expression, Sweet orange, *huanglongbing*, Plant-pathogen interaction

## Abstract

**Background:**

Citrus *huanglongbing* (HLB) disease is caused by endogenous, phloem-restricted, Gram negative, uncultured bacteria named *Candidatus* Liberibacter africanus (CaLaf), *Ca.* L. asiaticus (CaLas), and *Ca.* L. americanus (CaLam), depending on the continent where the bacteria were first detected. The Asian citrus psyllid vector, *Diaphorina citri*, transmits CaLas and CaLam and both Liberibacter species are present in Brazil. Several studies of the transcriptional response of citrus plants manifesting HLB symptoms have been reported, but only for CaLas infection. This study evaluated the transcriptional reprogramming of a susceptible genotype of sweet orange challenged with CaLam, using a customized 385K microarray containing approximately 32,000 unigene transcripts. We analyzed global changes in gene expression of CaLam-infected leaves of sweet orange during the symptomatic stage of infection and compared the results with previously published microarray studies that used CaLas-infected plants. Twenty candidate genes were selected to validate the expression profiles in symptomatic and asymptomatic PCR-positive leaves infected with CaLas or CaLam.

**Results:**

The microarray analysis identified 633 differentially expressed genes during the symptomatic stage of CaLam infection. Among them, 418 (66%) were upregulated and 215 (34%) were down regulated. Five hundred and fourteen genes (81%) were orthologs of genes from *Arabidopsis thaliana*. Gene set enrichment analysis (GSEA) revealed that several of the transcripts encoded transporters associated with the endomembrane system, especially zinc transport. Among the most biologically relevant gene transcripts in GSEA were those related to signaling, metabolism and/or stimulus to hormones, genes responding to stress and pathogenesis, biosynthesis of secondary metabolites, oxidative stress and transcription factors belonging to different families. Real time PCR of 20 candidate genes validated the expression pattern of some genes in symptomatic and asymptomatic leaves infected with CaLam or CaLas.

**Conclusions:**

Many gene transcripts and biological processes are significantly altered upon CaLam infection. Some of them had been identified in response to CaLas infection, while others had not been previously reported. These data will be useful for selecting target genes for genetic engineering to control HLB.

## Background

*Huanglongbing* (HLB) is considered a devastating citrus disease and affects most of the main citrus growing areas worldwide. It has become a serious threat to Brazil and USA, the largest producers of sweet orange and frozen concentrated orange juice, since the first reports of the disease in 2004 [1] and 2005 [2], respectively. In Brazil, the causal agents of HLB are the Gram-negative and phloem-restricted bacteria *Candidatus* Liberibacter asiaticus (CaLas) and *Ca*. L. americanus (CaLam). Another species, *Ca*. L. africanus (CaLaf), occurs in Africa and is not found in South, Central or North America. Both CaLas and CaLam are transmitted by the Asian citrus psyllid (*Diaphorina citri*); however, they can also be transmitted with different levels of efficiency by grafting of infected tissues [3,4].

Once a citrus tree is infected, the time taken for HLB symptoms to appear varies according to the genotype; the physiological stage and age of the plant; the tissue used as the source of bacteria or the intensity of inoculation by the psyllid; and the season of the year. In leaves, typical symptoms include yellowing, asymmetric blotchy mottling and nutrient deficiency. Small, poor quality and lopsided fruits are frequently observed [5]. There is a clear disturbance of the transport system between source (leaves) and sinks (meristems, fruits, roots), with starch accumulation in plastids, chloroplast disruption, necrotic phloem (probably associated with plugging of sieve cells via callose deposition) and phloem-proteins accumulation [6-8]. Ultrastructural changes in the phloem and adjacent tissues can be observed in plants infected with CaLas, from the early (asymptomatic) stages of infection (three months), progressing to phloem degeneration nine months later [8]. However, before the complete degeneration of the phloem vessels, the bacteria move to newly growing tissues to restart the infection process [8]. Compared with CaLas, CaLam is less effectively transmitted both by vector and by grafting, is more sensitive to higher temperatures and reaches lower titer levels under the same experimental conditions. Both bacteria induce indistinguishable HLB symptoms in susceptible genotypes [9].

Using two different metagenomic approaches, two research groups reported the full genome of CaLas [10,11]. The authors showed that the bacterium has a small (1.23 Mb) genome, limited capacity for aerobic respiration and depends on several primary metabolites. It lacks type III and type IV secretion systems, avirulence factors, and degradative enzymes, which probably avoid the elicitation of defense response based on the products of plant cell wall degradation. However, the authors did not discount the possibility that CaLas may elicit a plant response mediated by a pathogen-associated molecular patterns (PAMP) and/or secrete an unknown effector that suppresses the host defense [11]. Thus, together with the absence of important housekeeping genes associated with the difficulty to culture the bacterium in artificial medium [12,13], its capacity to multiply both in plants and insects, and its intracellular habitat in the phloem sieve tubes suggest a parasitic, rather than a pathogenic, lifestyle. Additionally, two highly related, circular prophages have been found integrated into the CaLas genome [14]. One of them, SC1, is apparently fully functional, with a lytic cycle that appears to be activated in plants, but not in psyllids. The other prophage, SC2, is apparently in the lysogenic phase both in plants and psyllid infected by CaLas. Whether or not the CaLas prophages play a direct role in its competitiveness with CaLam remains to be determined; however, Zhang et al. suggested that this possibility could not be ruled out [14]. Taken together, these features clearly suggest an improved fitness of CaLas when compared with CaLam, and may explain why, in recent years, this species has become prevalent in Brazil. Nevertheless, CaLam is still found in the field and there is at least one report of this species in China [15].

Several studies on the transcriptional response of citrus plants showing HLB symptoms have been reported; however, all of them focused on CaLas infection. Overall, they show complex gene expression modulation in response to CaLas, but no specific mechanism associated with the infection has been identified yet [6,16-20]. It should be noted that the studies have focused on CaLas because of its worldwide importance; however, finding common features - and differences - between the two species, and differences in the response of citrus to them, may lead to a better understanding of the pathogenesis of HLB and how citrus plants respond to the disease. The present study evaluated the transcriptional reprogramming of leaf tissue of sweet orange during the symptomatic stage of infection with CaLam, using a robust, customized, 385K-microarray chip containing approximately 32,000 unigenes from *Citrus sinensis* L. Osb. A set of candidate genes from this and from previous studies was used to validate the expression profile in symptomatic and asymptomatic PCR-positive leaf tissue of sweet orange infected with CaLas or CaLam. Based on these results, we described the main alterations in gene expression during the late stages of infection of CaLam compared with CaLas and discussed how this information could be useful for genetic engineering to control HLB.

## Results

### Detection of the bacteria

Pera sweet orange plants were grafted with two CaLam-infected buds and the bacterium was first detected by PCR only 56 weeks after grafting (wag). Plants displayed typical symptoms of asymmetrical blotchy mottling of leaves at 80 wag. Hamlin sweet orange grafted either with two CaLam- or CaLas-infected buds were PCR positive at 64 and 32 wag, respectively. Symptoms of asymmetrical blotchy mottling of leaves were detected in plants inoculated with CaLas at 40 wag, whereas plants inoculated with CaLam displayed symptoms only at 104 wag. Both the initial bacterial titers in the source buds, the differences between the efficiency of graft transmission and differences in the multiplication of the bacterium inside the plant could explain the discrepancies between the times of incubation and development of symptoms [3].

### Overview of changes in gene expression in response to CaLam infection

Microarray analysis identified 633 differentially expressed genes (DEGs) in symptomatic leaves infected with CaLam: 418 of them (66%) were upregulated and 215 (34%) were downregulated (p-values ≤0.05, fold change |FC| ≥ 2.0 and odds probability ≥ 0.95). Among these DEGs, 514 (81%) were identified as orthologs of *A. thaliana* genes (Additional file 1). Although microarray experiments were performed using only two replicates, correlations within groups were very high (approximately 97% for both symptomatic and controls). To determine which genes and pathways were relevant during the symptomatic stage of CaLam infection, a GSEA approach was used. Based on GSEA, a hypergeometric test (p-values <0.005) was applied to identify which cellular components (CCs), molecular functions (MFs) and biological processes (BPs) were overrepresented in our ordered list of DEGs.

GSEA revealed many DEGs putatively associated with the endomembrane system, apoplast and chloroplasts (Additional file 2). These genes were mainly involved with the photosynthetic apparatus and the expression level of many of them was downregulated in symptomatic plants compared with control plants. Among the genes associated with the endomembrane system, transcripts encoding three zinc transporters (ZIP1, ZIP4 and ZIP5) and a zinc inducer facilitator (ZIF1), which is involved in Zn homeostasis and Zn sequestration, were upregulated.

With regard to MFs, the DEGs were mainly associated with catalytic activity, transcription regulation and transport of specific substrates and metabolites (Additional file 2). In addition to the Zn transporters, several transcripts encoding auxin transporters were differentially expressed in symptomatic plants compared with control plants.

**Figure 1:**
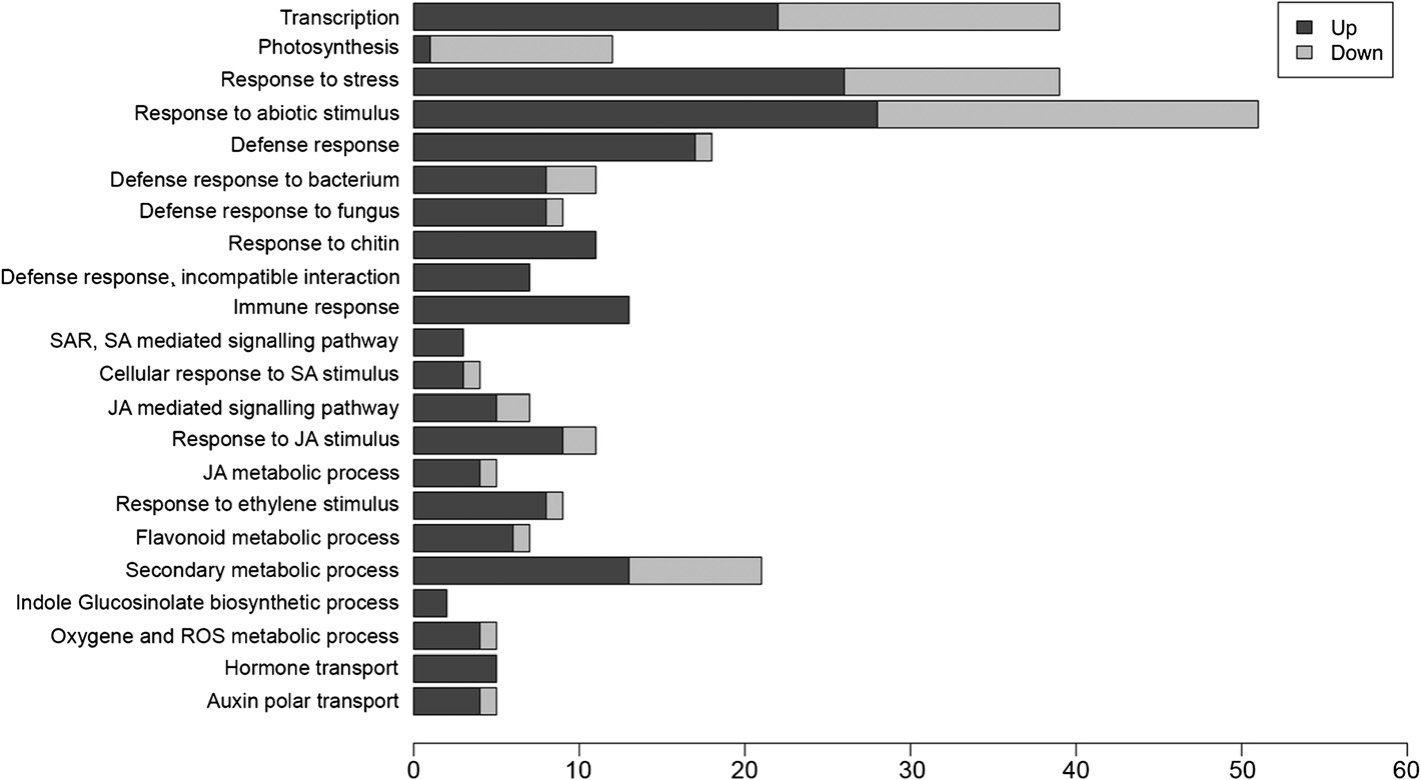
**Distribution of differentially expressed genes (DEGs) (x-axis) into Gene Ontology (GO) categories (biological process) (y-axis) according to Gene Set Enrichment Analysis (GSEA).** Only biological processes (BPs) discussed in the results are presented here. A complete list of BPs can be found in Additional file 2.

Furthermore, GSEA revealed significant BPs altered in symptomatic plants upon infection with CaLam. We found that many BPs related to hormone signaling and metabolism, stress response, photosynthesis, secondary metabolism and transcription were overrepresented by GSEA. Moreover, several specific BPs associated with defense against biotic stress, such as immune response, incompatible interactions, systemic acquired resistance, oxidative stress and response to bacteria were identified (Figure 1). A complete list of DEGs selected from the enrichment analyses can be found in Additional files 3 and 4.

### Main processes or pathways affected by the response to infection with CaLam

#### Carbohydrate metabolism

Carbohydrates are the most abundant translocated solutes in the phloem, and sucrose is the predominant sugar found in the phloem sieve tubes [21]. In our analysis, CaLam affected the expression patterns of several genes related to sugar transport, metabolism and degradation of starch (Additional file 1). Notably, the glucose-6-phosphate/phosphate transporter 2 (GPT2) was the most strongly regulated transcript in CaLam symptomatic, infected leaves. Moreover, we found differentially expressed transcripts encoding other enzymes related to biosynthesis and/or metabolism of galactose and raffinoses (RFOs) and several UDP-glucuronosyltransferases (UGTs).

#### Genes involved with phloem functionality and callose deposition

CaLam infection modulated several genes with important functions in the phloem (Additional file 1). Among the induced transcripts, we found those encoding a phloem protein-2 (PP2*-*B10), a PP2-B14 and a homogentisate phytyltransferase (VTE2); the latter is involved in tocopherol biosynthesis and phloem sucrose loading. Moreover, a transcript encoding a cytochrome P450 (CYP83B1), an enzyme involved in the biosynthesis of glucosinolates and defense response by callose deposition, was also upregulated in symptomatic leaves infected with CaLam [22].

#### Transcription factors (TF)

Reprogramming of gene expression upon CaLam infection is regulated by many transcription factors. Among the 38 differentially expressed transcription factors, MYB, ethylene response factors (ERF), bHLH and WRKY family proteins were the most abundant. A myb-like gene transcript was the most induced transcription factor: it was approximately 100-fold upregulated in symptomatic compared to control plants (Additional file 1).

#### Defense and pathogenesis related genes

According the GSEA, several functional categories were found (Figure 1). Thirty-nine transcripts were found in the category “response to stress” (GO: 0006950) and 18 of them were also annotated with the GO term “defense response” (GO: 0006952). Among them, transcripts encoding a receptor-like protein (RLP12), two pathogenesis-related protein (PR) (PR3 and PR4), an osmotin-like protein (OSM34) and transcripts for a U-box type E3 ubiquitin-ligase (PUB22) were the most induced. Expression levels of such genes increased in symptomatic leaves by 10- to 84-fold compared with the leaves of control plants. Other DEGs were moderately induced, such as several nucleotide-binding site–leucine-rich repeat (NBS-LRR) genes, some gene transcripts belonging to the salicylic acid signaling pathway, a LysM receptor like kinase (CERK1) and other receptor-like proteins (RLPs). Only six transcripts were downregulated in symptomatic plants, among them there were a salicylic acid-binding protein 3 (SABP3) and a beta carbonic anhydrase 2 (CA2), which are both related to the defense response to bacteria (Table 1). The complete list of DEGs and their related biological processes are shown in Additional files 3 and 4.

**Table 1:** Subset of differentially expressed genes (DEGs) in leaves of symptomatic sweet orange plants infected with CaLam compared with non-infected plants

**Probe set**	**Gene symbol**	**Annotation**	**TAIR code**	**Fold change**	**Adjusted P-value**	**Odds probability**
*GO:0042742 - Defense response to bacterium*						
101776	CSD1	Cytosolic copper/zinc superoxide dismutase	AT1G08830	73.5	8.04E-10	1.00E + 00
120012	CSD1	Cytosolic copper/zinc superoxide dismutase	AT1G08830	64.0	2.47E-06	1.00E + 00
111286	OSM34	Osmotin-like protein	AT4G11650	17.8	3.12E-01	1.00E + 00
119264	WRKY40	Pathogen-induced transcription factor	AT1G80840	16.8	9.25E-02	1.00E + 00
106985	WRKY40	Pathogen-induced transcription factor	AT1G80840	5.6	8.80E-01	1.00E + 00
119954	WRKY40	Pathogen-induced transcription factor	AT1G80840	5.4	8.04E-04	9.55E-01
108464	JAZ1	Similar to unknown protein [*Arabidopsis thaliana*] (TAIR:AT1G74950.1)	AT1G19180	7.4	9.67E-01	1.00E + 00
114567	JAZ1	Similar to unknown protein [*Arabidopsis thaliana*] (TAIR:AT1G74950.1)	AT1G19180	4.6	2.98E-04	9.88E-01
109695	JAZ1	Similar to unknown protein [*Arabidopsis thaliana*] (TAIR:AT1G74950.1)	AT1G19180	4.3	2.32E-04	9.91E-01
104101	WRKY70	Member of WRKY Transcription Factor; Group III	AT3G56400	5.1	8.62E + 00	9.97E-01
118920	CYP83B1	Oxime-metabolizing enzyme	AT4G31500	4.8	3.20E-01	1.00E + 00
115708	CYP83B1	Oxime-metabolizing enzyme	AT4G31500	4.3	2.03E-04	9.93E-01
103563	PAD4	Lipase-like gene	AT3G52430	3.7	4.91E + 00	9.99E-01
119801	NHL3	Similar to tobacco hairpin-induced gene (HIN1) and Arabidopsis NDR1	AT5G06320	3.4	4.07E-04	9.82E-01
125507	SABP3	Carbonic anhydrase 1	AT3G01500	-2.7	7.39E-04	9.60E-01
117969	SABP3	Carbonic anhydrase 1	AT3G01500	-2.8	1.29E-04	9.96E-01
104171	SABP3	Carbonic anhydrase 1	AT3G01500	-2.8	8.72E-04	9.50E-01
103796	SABP3	Carbonic anhydrase 1	AT3G01500	-3.9	2.28E + 00	1.00E + 00
114665	CA2	Beta carbonic anhydrase	AT5G14740	-4.0	3.38E + 00	9.99E-01
125402	PSII-P	Encodes a 23 kD extrinsic protein	AT1G06680	-3.1	4.11E-04	9.81E-01
115962	PSII-P	Encodes a 23 kD extrinsic protein	AT1G06680	-4.4	5.75E-04	9.71E-01
*Other ontologies related to defense response*						
125394	NA	Protease inhibitor, putative	AT2G38870	55.2	1.07E-01	1.00E + 00
119807	PUB22	U-box domain-containing protein	AT3G52450	45.2	3.35E-04	1.00E + 00
108272	PR3	Basic chitinase	AT3G12500	36.2	1.12E + 00	1.00E + 00
106567	PR3	Basic chitinase	AT3G12500	29.0	1.82E-04	9.93E-01
105539	PR3	Basic chitinase	AT3G12500	20.0	7.42E + 00	9.98E-01
113952	NA	Protease inhibitor, putative	AT2G38870	13.9	9.61E-02	1.00E + 00
113416	BSMT1	SABATH methyltransferase	AT3G11480	12.9	1.14E-01	1.00E + 00
118060	PR4	Similar to the antifungal chitin-binding protein	AT3G04720	12.0	2.08E + 00	1.00E + 00
117867	RLP12	Disease resistance family protein / LRR family protein	AT1G71400	10.9	1.13E-04	9.96E-01
113207	NA	Protease inhibitor, putative	AT2G38870	8.7	1.98E-01	1.00E + 00
111466	ANNAT4	Annexins	AT2G38750	8.6	8.00E + 00	9.98E-01
131326	EP3	EP3 chitinase	AT3G54420	8.3	1.05E-02	1.00E + 00
118304	ANNAT4	Annexins	AT2G38750	8.0	8.21E + 00	9.98E-01
117348	CC-NBS-LRR	Disease resistance protein (CC-NBS-LRR class), putative	AT1G50180	7. 5	9.63E-01	1.00E + 00
117318	NBS-LRR	Disease resistance protein (NBS-LRR class), putative	AT3G14470	6.7	8.61E-01	1.00E + 00
112603	MLP423	Bet v I allergen family protein	AT1G24020	6.7	8.27E-04	9.53E-01
116735	VTE2	Homogentisate phytyltransferase	AT2G18950	6.4	7.08E-04	9.62E-01
118180	VTE2	Homogentisate phytyltransferase	AT2G18950	5.9	6.88E-04	9.64E-01
111673	CERK1	Protein kinase family protein	AT3G21630	5.9	5.58E + 00	9.99E-01
118780	VTE2	Homogentisate phytyltransferase	AT2G18950	5.4	8.09E-04	9.55E-01
107982	WRKY6	transcription factor WRKY6 (WRKY6)	AT1G62300	5.3	4.03E + 00	9.99E-01
102976	NBS-LRR	Disease resistance protein (NBS-LRR class), putative	AT3G14470	5.2	8.83E-01	1.00E + 00
125093	ERF-1	Ethylene response factor subfamily B-3 of ERF/AP2 transcription factor family	AT4G17500	5.1	1.17E-04	9.96E-01
118139	NBS-LRR	Disease resistance protein (NBS-LRR class), putative	AT3G14470	5.1	3.12E-01	1.00E + 00
101619	ERF1	Ethylene response factor subfamily B-3 of ERF/AP2 transcription factor family	AT3G23240	4.8	6.40E-04	9.67E-01
118745	RLP35	Disease resistance family protein	AT3G11080	4.2	7.30E-01	1.00E + 00
110958	CC-NBS-LRR	Disease resistance protein (CC-NBS-LRR class), putative	AT1G50180	4.1	4.74E-01	1.00E + 00
123922	MYB44	Member of the R2R3 factor gene family	AT5G67300	4.1	1.77E-04	9.94E-01
127726	PUB29	U-box domain-containing protein	AT3G18710	3.8	3.30E-04	9.86E-01
123451	RLP52	Putative disease resistance protein induced by chitin oligomers	AT5G25910	3.3	2.16E + 00	1.00E + 00
100053	EDA39	EDA39 (embryo sac development arrest 39); calmodulin binding	AT4G33050	3.3	3.30E-04	9.86E-01
120034	NBS-LRR	Disease resistance protein (NBS-LRR class), putative	AT3G14460	3.2	2.27E-04	9.91E-01
109138	NA	Ethylene response factor subfamily B-4 of ERF/AP2 transcription factor family	AT5G61890	3.1	3.84E-04	9.83E-01
111503	RBOHD	NADPH/respiratory burst oxidase protein D	AT5G47910	2.7	1.98E-04	9.93E-01
109625	RLP7	Disease resistance family protein	AT1G47890	2.7	6.78E-04	9.64E-01
125586	EDS1	Component of R gene-mediated disease resistance	AT3G48090	2.5	8.09E-04	9.55E-01
118211	TIR-NBS-LRR	Disease resistance protein (TIR-NBS-LRR class), putative	AT4G12010	2.5	8.19E-04	9.54E-01
105065	AN1-like	Zinc finger (AN1-like) family protein	AT3G52800	2.4	4.11E-04	9.81E-01
103141	RLP54	Disease resistance family protein	AT5G40170	2.3	4.40E-04	9.79E-01
100163	CIPK25	Member of AtCIPKs	AT5G25110	-3.3	1.50E-04	9.95E-01
109438	ST	High similarity to flavonol sulfotransferases (FSTs)	AT2G03760	-7.9	8.62E-02	1.00E + 00
131275	NA	Disease resistance-responsive protein-related / dirigent protein-related	AT2G21100	-10.9	1.28E + 00	1.00E + 00

Only DEGs annotated in the Defense response (GO:0006952) biological process and/or child ontologies related to defense are shown. The complete list of DEGs is shown in Additional file 1.

#### Oxidative stress and detoxification

The most upregulated transcript in the group of genes responding to oxidative stress was that encoding a cytosolic copper/zinc superoxide dismutase (CSD1), which was expressed at a 73-fold higher level in symptomatic plants compared with the control. CSD1 is an important superoxide dismutase involved in detoxification of superoxide radicals. Intriguingly, a chloroplastic copper/zinc superoxide dismutase (CSD2), which has the same function as CSD1, was downregulated. By contrast, transcripts for key enzymes related to reduction of reactive oxygen species (ROS) were induced, although only slightly, including a respiratory burst oxidative homolog D (RbohD) gene, which encodes an enzyme implicated in the generation of ROS during the defense response [23].

#### Secondary metabolism

Among the most relevant BPs identified in GSEA were those related to secondary metabolic processes, phenylpropanoid biosynthetic processes and biosynthesis and metabolism of flavonoids and indole glucosinolates. Among the differentially expressed genes, transcripts encoding three oxidorreductase 2OG-Fe(II) oxygenases were upregulated. Other differentially modulated transcripts in symptomatic citrus that are related to secondary metabolism are listed in Additional files 3 and 4.

### Real time quantitative PCR (RT-qPCR) assays to validate candidate genes

**Figure 2:**
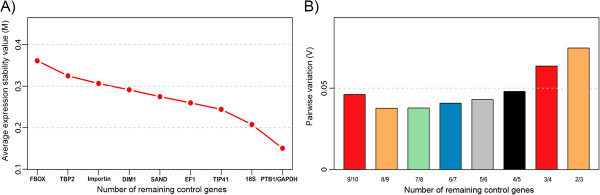
**Average expression stability values (M) and Pairwise variation (V) of the ten citrus reference genes calculated by geNorm. A)** A lower M value indicates more stable expression. **B)** Pairwise variation (V) to determine the optimal number of reference genes suggested by geNorm to a reliable normalization.

Before RT-qPCR analysis, we tested the expression stability of ten reference genes (F-box, TBP2, PTB1, Importin, 18S ribosomal, EF1α, SAND, GAPDH, DIM1 and TIP41) to find the best pair of genes for normalizing the expression levels of the candidate genes. Using geNorm, we defined PTB1 and GAPDH as the most stable pair of reference genes for RT-qPCR (Figure 2A). Pairwise variation analysis revealed that PTB1 and GAPDH should be sufficient for a reliable normalization (Figure 2B).

**Figure 3:**
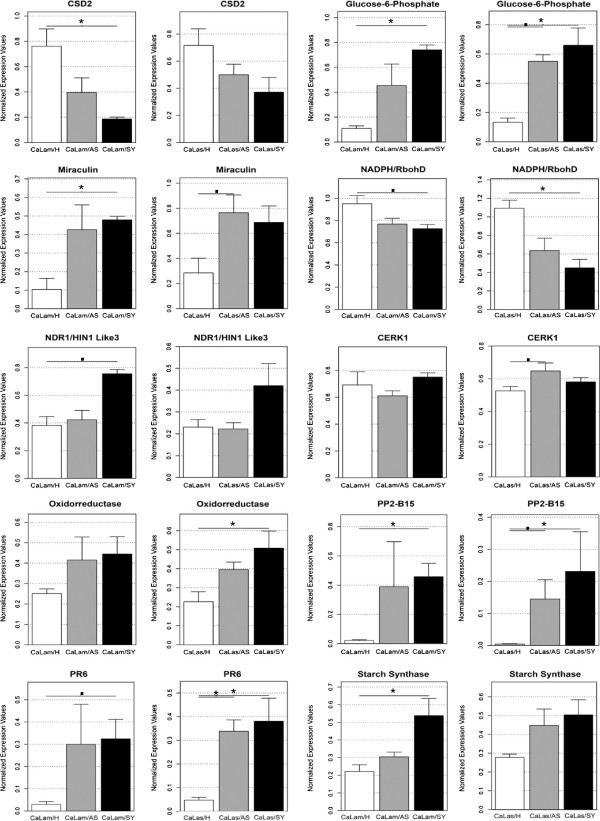
**Comparison of the expression levels of a subset of ten genes in symptomatic (SY) and asymptomatic (AS) leaves of Hamlin sweet orange infected with CaLas or CaLam in relation to their controls (H) by RT-qPCR.** Comparisons were performed by a nonparametric one-way ANOVA with 1000 unrestricted permutations, followed by pair-wise comparisons with Bonferroni adjustment. Levels of significance less than or equal to 0.05 and 0.1 were considered as “significant” (*) and “suggestive” (^.^), respectively. The remaining ten genes tested by RT-qPCR can be found in Additional file 5.

Among the 20 genes tested by RT-qPCR, transcripts for eight and five genes were statistically differentially expressed only in leaves challenged with CaLam and CaLas in relation to their controls, respectively (Figure 3 and Additional file 5). Transcripts for five genes were differentially modulated either in response to CaLam or to CaLas challenging. Four of them (GPT2, miraculin, PP2-B15 and PR6) were upregulated (Figure 3), whereas transcripts for NADPH/ RbohD were downregulated in comparison with controls when assayed by RT-qPCR (Additional file 5). Two gene transcripts (SABP3 and USP) showed a non-statistically significant trend for differential modulation in symptomatic or asymptomatic leaves (both infected with CaLam or CaLas) in relation to the control, according to the RT-qPCR assays (Additional file 5). These results differed from those obtained by the microarray, in which transcripts for a SABP3 gene were downregulated, whereas transcripts for USP were slightly induced during the symptomatic phase of CaLam infection compared with the control.

In terms of the differential expression during the asymptomatic or symptomatic stage of infection, some differences were found depending on the bacterium species used. In general, few genes expressions were differentially modulated during the asymptomatic phase of CaLam or CaLas infection. In the asymptomatic leaves infected with CaLam, the expression of two genes (auxin efflux carrier and VTE2) were induced and repressed, respectively, compared with the control (Additional file 5).

During the symptomatic phase of CaLam infection, transcripts for 12 genes were differentially expressed: auxin efflux carrier, PP2-B10, RLP7 and Kunitz family protein (Additional file 5); CSD2, GPT2, miraculin, NADPH/RbohD, NDR1/HIN1-like 3, PP2-B15, PR6 and starch synthase (Figure 3). Among these 12 gene transcripts, nine of them showed similar expression patterns by RT-qPCR compared with the microarray: starch synthase, CSD2, GPT2, Kunitz family protein, NDR1/HIN1-like 3 and PR6 (Figure 3) and PP2-B10, RLP7 and auxin efflux carrier (Additional file 5). Transcripts for a homogentisate phytyltransferase (VTE2) showed a significant reduction in expression only in asymptomatic compared to control plants (Additional file 5) and transcripts for a NADPH/ RbohD, whose expression was observed to slightly increase in the microarray analysis, showed a decrease in expression level when assayed by RT-qPCR (Figure 3). The phloem-protein B15 (PP2-B15), which is reported as one of the most upregulated genes in response to CaLas [6,16,17], also showed a progressive increase in expression in asymptomatic and symptomatic leaves infected with CaLam. Unlike CaLam, the expression of six genes was altered during the asymptomatic phase of CaLas infection: GPT2, miraculin, CERK1, PP2-B15, PR6 (Figure 3) and WRKY70 (Additional file 5). Three of them were also differentially expressed in symptomatic leaves: GPT2, PP2-B15 and PR6 (Figure 3). In addition, transcripts for NADPH/RbohD, oxidorreductases (Figure 3), WRKY25 and PR1 (Additional file 5), were differentially expressed only during the symptomatic stage of CaLas infection.

Discrepancies between the expression levels of DEGs identified in the microarray and by RT-qPCR could be related to technical differences in the sensitivity and specificity between the methods, and biological variations, i.e., differences between the genotypes used.

## Discussion

HLB is considered the most destructive citrus disease worldwide, and in Brazil it is caused by CaLam and CaLas. CaLas and CaLam are transmitted by the Asian citrus psyllid and are restricted to the phloem of infected citrus, where they can multiply and spread, causing a severe imbalance in the translocation of nutrients and other important metabolites. Phloem is the main trafficking pathway of nutrients, defensive compounds and signaling molecules throughout the plant; thus, several relevant biological processes are affected in citrus infected with Liberibacters.

Our study aimed to investigate the transcriptome reprogramming of citrus upon infection with CaLam. Microarray analysis identified 514 DEGs, which were grouped into gene ontology (GO) categories and ranked according to the most representative GO terms, as calculated by the GSEA approach. Among the molecular functions overrepresented, we found that gene transcripts encoding three zinc transporters (ZIP1, ZIP4 and ZIP5) were overexpressed. High levels of ZIP1 transcripts (FC = 30.48) were also observed in the transcriptome of ‘Navel’ and ‘Madam Vinous’ sweet oranges infected with CaLas, although ZIP1 was not found among the proteins evaluated in the citrus proteome [19]. Similarly, transcripts for a ZIP5 transporter were highly induced in the infected susceptible genotype (Cleopatra mandarin) but not in the tolerant genotype (US-897) [17]. The overexpression of transcripts for Zn transporters in citrus in the late stages of infection with CaLam or CaLas are expected, because the symptoms in the leaves of susceptible citrus plants often resemble those of zinc deficiency. In fact, in some citrus genotypes, the concentrations of Zn and Fe in infected plants was found to be approximately half those in healthy plants, which indicates that zinc homoeostasis is significantly affected during infection [24]. As an important micronutrient in plants, zinc has structural (e.g., carbonic anhydrase) and catalytic functions (e.g., superoxide dismutase CuZn-SOD) in many of enzymes. In addition, zinc is involved in the maintenance of membrane integrity and protection of cell structural components against the oxidative damage caused by ROS. As a result, several studies demonstrated that an imbalance in the intracellular zinc concentration affects not only optimal plant growth, but also, in some cases, the susceptibility/tolerance of plants to certain pathogens [25,26]. In susceptible citrus infected with Liberibacters, zinc deficiency has, to some extent, been associated with inefficient translocation of this mineral by the roots. An important issue to be addressed is whether Liberibacters could directly or indirectly reduce the availability of this metal as a strategy to favor the infection process [24], as observed for *Xanthomonas oryzae* in rice [27].

In addition to nutritional deficiencies observed during symptom progression of HLB, several studies reported that CaLas infection dramatically affects carbohydrate metabolism [8,17]. The imbalance of carbohydrate partitioning causes an accumulation of starch in infected leaves during the progression of HLB symptoms. Evidence for this observation include increases in the starch content in symptomatic leaves, microscopic observations of starch accumulation in phloem parenchyma cells of infected leaves, induction of transcripts encoding enzymes related to starch biosynthesis (such as ADP-glucose pyrophosphorylase (AGPase)) and repression of transcripts related to starch breakdown [6,16,17,19]. Similarly to CaLas infected leaves, we found an induction of transcripts encoding key enzymes involved in starch biosynthesis and repression of those related to starch breakdown in CaLam infected leaves. Real time PCR of the starch synthase gene confirmed its upregulation in symptomatic leaves infected with CaLam and showed increased expression from asymptomatic to symptomatic leaves (Figure 3). High levels of sucrose and glucose in symptomatic leaves infected with CaLas have also been documented [16,19]. The increase in glucose levels explains the significant induction of transcripts for glucose-6-phosphate/phosphate transporter 2 (GPT2) in the transcriptome of citrus leaves infected with CaLas [17]. In our microarray analysis, GPT2 was also significantly induced in symptomatic leaves infected with CaLam compared with the control. RT-qPCR analysis showed a significantly increased expression of transcripts for GPT2 in symptomatic and asymptomatic leaves infected with both CaLas and CaLam (Figure 3).

Transcripts encoding enzymes related to raffinose metabolism, another class of sugars found in phloem sap, were also modulated during CaLam infection. Among them, a galactinol synthase (GLS8) increased by seven-fold in infected plants. Galactinol synthase is the first enzyme in the synthesis of RFOs and regulates the partitioning between sucrose and RFOs, whereas raffinose synthases catalyze the synthesis of RFOs from sucrose and galactinol. High intracellular levels of RFOs have been correlated with osmoprotection in plant cells; however, recent studies reported RFOs as potential scavengers of ROS, suggesting a novel role for RFOs in protection against oxidative stress [28]. We hypothesized that the induction of enzymes involved in the biosynthesis of RFOs may be an attempt to reduce the level of sucrose in the phloem of infected leaves, although this strategy does not appear to be effective in preventing the accumulation of starch in leaves and the subsequent consequences on the translocation of nutrients from source to sink organs. Furthermore, RFOs could also function as potent antioxidants to minimize the oxidative stress that occurs near to the necrotic sieve elements formed during CaLam infection.

Transcripts encoding three UDP-glucosyltransferases (UGT84A1; UGT76B1 and a putative UGT) were overexpressed in symptomatic leaves upon infection with CaLam. UGTs catalyze the transfer of glucosyl residues from UDP-glucose to a wide range of secondary metabolites and hormones, such as salicylic acid (SA). A transcript for UGT76B1, which was slightly induced in our microarray analysis, has been reported as a key player in the crosstalk between SA-jasmonic acid (JA) signaling. The knockout of UGT76B1 in *Arabidopsis* led to enhanced resistance to *Pseudomonas syringae*, but an increased susceptibility to *Alternaria brassicicola*[29]. In HLB-infected citrus trees, transcripts encoding different UGTs were modulated in leaves [17] and in fruit tissues infected with CaLas [18]. Among them, transcripts encoding a UGT73B3 were more abundant in infected leaves of the tolerant hybrid compared with Cleopatra, the susceptible citrus genotype. Unlike UGT76B1, knockout of UGT73B3 and its homolog UGT73B5 in *Arabidopsis* led to increased susceptibility to *Pseudomonas syringae*[30]. The modulation of several UGTs either in CaLam or CaLas infected plants reveals the potential application of UGTs as target genes for genetic engineering.

During the symptomatic stage of HLB, the deposition of callose and P-proteins has been observed in the sieve pores of the sieve elements of the leaf phloem [8,16]. P-proteins are structural proteins involved in sealing off damaged sieve elements by plugging up the sieve plate pores. This dynamic and reversible mechanism is frequently accompanied by a long-term solution to sieve tube damage: callose deposition in the sieve pores. Several transcriptome studies of citrus infected with CaLas have reported a strong induction of transcripts encoding the phloem protein PP2-B15 in symptomatic leaves of sweet oranges compared with the control [6,16,17]. In our microarray analysis, we found transcripts encoding two different phloem proteins, PP2-B10, which was highly induced and PP2-B14, which was moderately induced. We tested the expression of PP2-B10 by RT-qPCR and confirmed an increase in the level of this transcript in asymptomatic and symptomatic CaLam infected leaves compared with control leaves (Figure 3). PP2-B15 was also assayed by RT-qPCR and showed a similar pattern of overexpression found to PP2-B10 in symptomatic and asymptomatic leaves infected with CaLam. In relation to CaLas, our RT-PCR assays confirmed an induction of PP2-B15 during infection, but PP2-B10 was not significantly modulated. With regard to callose deposition, we found that transcripts encoding a cytochrome P450 (CYP83B1), an enzyme involved in glucosinolate biosynthesis, were four-fold induced in the microarray analysis. Transcripts encoding a CYP83B1 were upregulated in symptomatic flavedo of citrus fruits upon CaLas infection [18]. Indole glucosinolates (IGs) are secondary metabolites derived from tryptophan, which have a well-characterized role in insect resistance [31]. However, Clay and co-workers reported that IGs and their breakdown products were required for callose deposition, which was effective in restricting *Pseudomonas syringae* growth in *Arabidopsis*[22]. According to the microarray analysis, transcripts encoding a homogentisate phytyltransferase (VTE2), the first enzyme of the tocopherol biosynthetic pathway, were also induced. Tocopherols are antioxidants that have roles in protecting chloroplast membranes and the photosynthetic apparatus from oxidative damage. However, studies have shown that tocopherol has an important role in regulating the phloem loading in low-temperature adaptation [32,33]. Intriguingly, loss of VTE2 function-mutants, which were deficient in tocopherol, exhibited an inhibition of photoassimilate transport, followed by an increase of solute sugar and consequently starch, and callose deposition in phloem parenchyma transfer cell walls adjacent to the companion cell/sieve element complex [33], leading to a phenotype that resembles HLB in citrus. Impairment of phloem loading is a major consequence of Liberibacter infection; therefore, modulation of tocopherol biosynthesis in citrus by overexpression of VTE2 during early infection could be an interesting approach for increasing the phloem translocation of nutrients and for minimizing the symptoms.

Considering that CaLam infection affected different biological processes in citrus, it is not surprising that the expression of many TFs were differentially modulated. Microarray analysis identified transcripts for 38 TFs that were differentially expressed in symptomatic leaves infected with CaLam. The most highly induced TF was a myb-like gene, which regulates the expression of several genes in response to phosphate during sucrose starvation in *Arabidopsis*[34,35]. In addition to having regulatory roles in the defense response upon infection with different pathogens [36-38], several MYB genes have been reported as key regulators of sugar-responsive genes, such as α-amylase during sugar starvation in rice [39]. Interestingly, the same myb-like gene was nearly 200-fold induced in symptomatic leaves of susceptible plants infected with CaLas, but not in the tolerant genotype, indicating that the upregulation of this gene could be associated with the susceptibility of citrus to *Ca.* Liberibacter spp. or, to some extent, to the manifestation of symptoms [17]. Whether this myb-like gene is involved in regulating the expression of defense response genes or sugar metabolism genes in response to CaLam and CaLas infection remains to be proven.

Among the differentially expressed defense-related gene transcripts in CaLam-infected citrus leaves were several for receptor-like proteins and a LysM receptor-like kinase (CERK1). Although the differential expression of transcripts encoding a CERK1 could not be confirmed by RT-qPCR in CaLam infected leaves, this gene was induced in asymptomatic leaves infected with CaLas. CERK1 is a receptor implicated in the perception of chitin, an essential component of the cell walls of all fungi, which acts as elicitor of the defense response in plants [40]. Despite the recognition of the fungal PAMP chitin by CERK1, a recent study showed that this receptor was able to recognize the bacterial type III effector protein, AvrPtoB [41]. Although bacteria do not contain chitin, other carbohydrates with similar structures to chitin, or even an unknown bacterial PAMP, could be potential ligands of the LysM domain of CERK1 [42,43]. CaLas does not have the type III secretion system or the degradative enzymes of type II [11]. However, it has been suggested that lipopolysaccharides (LPS) and LPS fragments, or even an unknown non-type III effector, could be the potential PAMP of CaLas [11]. In addition, we found that transcripts encoding three PR proteins (PR3, PR4 and PR6) were highly induced in our microarray data. PR3 and PR4 belong to the chitinase class, whereas PR6 proteins are defined as a subclass of serine proteinase inhibitors. Plant proteinase inhibitors play distinct physiological roles, including seed dormancy and protection against proteolytic enzymes of herbivores and pathogens. One of the first studies of the transcriptome of citrus leaves infected with CaLas reported an induction of PR6 only in asymptomatic leaves collected 5–9 weeks after inoculation [6]. In that paper, PR6 was annotated as a putative protease inhibitor (At2g38870). Our RT-qPCR data showed that both CaLas and CaLam induced PR6 in asymptomatic and symptomatic stages during infection. PR6 of *Arabidopsis* was induced in leaves upon infection with *Botrytis cinerea* and the resistance against this fungus was enhanced in transgenic lines overexpressing this gene [43]. To the best of our knowledge, few reports have demonstrated the induction of PR6 or other serine-proteinase inhibitors in plants upon infection with bacterial pathogens [44]. Further studies are needed to determine the role of PR6 in the Liberibacters *vs*. *Citrus* spp. interaction.

Among the gene transcripts moderately induced in the microarray analysis, we found an NDR1/HIN1-like3 (NHL3) gene. RT-qPCR showed a slight induction in the expression of this gene only in symptomatic leaves infected with CaLam. The expression of NHL3 was suppressed in *Arabidopsis* upon inoculation with a virulent *Pseudomonas syringae* (*Pst* DC3000); however, inoculation with a mutant *Pst* DC3000, which is deficient in the delivery of effector proteins of the Type III-secretion system, induced the expression of NHL3 [45].

Among the repressed gene transcripts related to defense response, we identified transcripts for two carbonic anhydrases: salicylic acid-binding protein 3 (SABP3) and beta carbonic anhydrase 2 (CA2). SABP3 has a high affinity for SA and its downregulation has been detected in CaLas infected leaves of citrus [6]. In addition, Slaymaker (2002) showed that in tobacco, SABP3 has antioxidant properties and has a role in the hypersensitive response [46]. Similarly, transcripts for CA2 were also reported to decrease significantly in leaves of *Arabidopsis* 12 hours post-inoculation with *Pseudomonas syringae*[47], which leads us to speculate that the downregulation of these genes could be associated with susceptibility of citrus to Liberibacters.

In addition to defense response genes, several transcripts encoding enzymes directly involved in oxidative stress were differentially modulated. Among them were transcripts for CSD1 and CSD2, which were induced and repressed, respectively, in symptomatic leaves infected by CaLam. This was also observed in transcriptome of citrus leaves upon infection with CaLas [6,17,19]. RT-qPCR analysis showed that transcripts encoding an NADPH oxidase (RbohD), which were slightly induced in the microarray analysis, were significantly repressed during both CaLam and CaLas infection (Figure 3). Increased expression of RbohD was responsible for accumulation of ROS during the defense response of *Arabidopsis* against *Pseudomonas syringae* and *Peronospora parasitica*[23]. In plants, ROS are continuously produced in mitochondria, chloroplasts, and peroxisomes as by-products of aerobic metabolic processes, such as respiration and photosynthesis. However, under stress conditions, the balance between production and scavenging of ROS is disturbed. During biotic stress, the production of ROS is termed the oxidative burst and is one of the first steps in the response of plants to pathogen attack. High levels of ROS can also be produced as a result of uncoupling, or inhibition, of the photosystem machinery in the chloroplast and during photorespiration [48]. It is not clear whether oxidative stress in late stages of HLB is part of a defense mechanism of citrus or a secondary effect caused by depletion of photosynthetic apparatus and necrosis of sieve elements. In any case, the role of the oxidative stress during CaLam or CaLam infection needs to be further investigated.

## Conclusions

This study aimed to unravel the changes in the transcriptome of symptomatic leaves of sweet orange upon infection with CaLam, using a customized 385K microarray chip containing about 32,000 *Citrus sinensis* cv Pera unigene transcripts. A large number of citrus transcripts and biological processes were significantly altered upon CaLam infection. Among the changes we highlight induction of transcripts for zinc transporters, differential modulation of transcripts encoding enzymes related to sugar metabolism, depletion of photosynthesis, induction of several defense-related genes, and modulation of transcripts encoding enzymes regulating ROS production. We found several biological processes differentially modulated in leaves during the symptomatic phase of CaLam infection, which are similarly affected by CaLas infection. Most of these biological processes probably reflect a secondary, rather than a primary, effect of the infection process. For example, the depletion of photosynthesis probably results from a mechanism of feedback inhibition caused by the accumulation of sucrose and glucose in leaves during infection [17]. Moreover, the impairment of the phloem vessels caused by deposition of P-proteins and callose appears to be a late and unsuccessful strategy of defense, because it does not prevent the bacteria from spreading through the plant. The blockage of the phloem vessels also affects the translocation of important nutrients through the plant. In this sense, PP2 gene silencing or silencing of genes related to the callose deposition would be a promising strategy to reduce the severity of symptoms of HLB, allowing the transport of nutrients through the phloem. However, the silencing of callose genes has been shown to favor the spread of *Xanthomonas citri* subsp*. citri*, (syn. *Xanthomonas axonopodis* pv. citri) leading to the development of the canker disease in citrus [49].

Microarray analysis showed several citrus transcripts that were differentially expressed in symptomatic compared to control plants are annotated as genes responsive to infection by bacterial pathogens, according to sequence homology to *Arabidopsis* genes. The identification of large number of transcripts coding for PR proteins, receptor-like proteins, NBS-LRR and transcription factors (such as WRKY and MYB) shows that even a susceptible citrus genotype is able to actively respond to infection by CaLam, as reported for CaLas. The development of HLB disease symptoms leads us to believe that the perception of the pathogen by the host and the subsequent activation or repression of genes involved in resistance is delayed or is insufficient to protect the plant from the pathogen. For this reason, certain defense-related genes that are able to increase the perception of the pathogen by the host and/or trigger a systemic defense response to CaLam and CaLas infection have been selected as candidates for citrus genetic engineering in our laboratory.

## Methods

### Challenge with *Ca.* Liberibacter

For the microarray analysis, biological experiments were set up in September 2008 (Spring), and performed with four-month-old shoot-tip grafted (virus-free) plants of Pera sweet orange grafted onto Rangpur lime (*C. limonia* L. Osb.). Firstly, plants were graft-inoculated using two buds from CaLam-infected Pera sweet orange trees kept in the greenhouse conditions and used as source of inoculum. Uninoculated plants (no mock) of the same age were maintained as control plants. All plants were kept in the greenhouse at a temperature ranging from 25 to 28°C, with a natural photoperiod and monitored bimonthly by end-point PCR [1] to detect the bacterium. Plants were inoculated again with one infected bud 32 weeks after the first grafting because of the low efficiency of grafting transmission of CaLam and the delay in bacterium detection and symptoms manifestation. Afterwards, all inoculated and control plants were then pruned and transferred to a growth chamber (Conviron) at 22 to 24°C, 16h/8h light/dark until the end of the experiment. Fully expanded leaves of two plants displaying symptoms of blotchy mottling and leaves of two healthy plants grown under the same conditions were collected separately, ground in liquid nitrogen, and stored at -80°C.

For real time PCR validation of the microarray analysis, a biological experiment was carried out in August 2010 using sweet orange Hamlin grafted onto Rangpur lime (*C. limonia* L. Osb.). Hamlin is an important sweet orange worldwide and, like Pera, is highly susceptible to the infection by CaLam or CaLas. To compare the differential expression of selected genes in response to the infection with different Liberibacter species, four-month-old shoot-tip grafted (virus-free) plants of Hamlin sweet orange were grafted with two buds from CaLam or CaLas infected sweet orange trees kept in the greenhouse conditions and used as source of inoculum. Uninoculated plants (no mock) of the same age were used as controls. All plants were kept in a greenhouse at a temperature ranging from 25 to 28°C, with a natural photoperiod and monitored bimonthly by end-point PCR to detect CaLam or CaLas. Fully expanded symptomatic (blotchy mottling) and asymptomatic (PCR positive) leaves of three plants, and healthy leaves of three control plants grown under the same conditions were collected separately, ground in liquid nitrogen, and stored at -80°C.

### Total RNA isolation and cDNA synthesis

Total RNA was extracted using an RNeasy Plant Mini Kit (QIAGEN, Valencia, CA, USA), according to the manufacturer’s instructions. Genomic DNA contamination was removed with recombinant DNAse I (QIAGEN). Total RNA concentration and purity were determined from the ratio of absorbance readings at 260 and 280 nm using a NanoDrop 8000 spectrophotometer (Thermo Fisher Scientific Inc., Wilmington, DE, USA), and RNA integrity was tested on a denaturing agarose gel. For RT-qPCR assays, reverse transcription was performed with 1 μg of total RNA in a total volume of 20 μL with oligo(dT) primer using RevertAid^TM^ H Minus First Strand cDNA Synthesis Kit (Fermentas, Burlington, ON, Canada). The final cDNA products were diluted 50-fold prior to use.

### Microarray experiment and data analysis

Roche Nimblegen Systems (Madison, WI, USA) designed an oligonucleotide array at a density of 385K. The customized chip comprised 31,541 unigene transcripts of *C. sinensis* cv Pera selected from the CitEST database, assembled from the expressed sequence tags (ESTs) submitted to NCBI (GenBank accession numbers EY649559 to EY842485)*.* Three probes for each unigene, with optimal predicted hybridization characteristics, were designed to comprise a probe set. Each probe set was then represented on the final array by four replicates. All probes were designed as perfectly matching oligonucleotides. Roche NimbleGen Systems carried out the array hybridization.

Raw data were imported to NimbleScan 2.5v software, which employs three steps of preprocessing: convolution background correction, quantile normalization [50] and a summarization of expression measures at the probe-level with a robust multiarray model fit (RMA) using the median polish algorithm [51]. Normalized expression values exported with the RMA.calls file extension were imported into R/BioConductor where statistical analyses were performed using Limma linear models [52]. Bayesian and moderated t-tests [53] were calculated and p-values were adjusted for multiple comparisons by the false discovery rate correction [54]. Unigene transcripts with p-values ≤0.05, fold change |FC| ≥ 2.0 and odds probability ≥ 0.95 were considered as DEGs. Unigene annotation was confirmed by BLASTX searches against the GenBank and TAIR databases, and further classified into categories according to the GO classification system. Microarray data can be accessed at the NCBI Gene Expression Omnibus (GEO) under accession GSE41221.

To identify relevant molecular mechanisms potentially associated with the response of sweet orange to CaLam infection, GSEA, which evaluates microarray data at the level of gene sets, was carried out [55]. A gene set was defined as all DEGs, with annotation according to *A. thaliana*, that share the same ontology based on the GO database. The GSEA method identified BPs, MFs and CCs that were overrepresented among a list of DEGs. The overrepresentation was assessed with a statistical score based on a hypergeometric test with p-values <0.005. Once detected, the potentially relevant processes revealed in our analysis were compared to those described in the previously published microarray analysis of citrus infected with CaLas [6,16,17,19]. Although the microarray platforms (Roche Nimblegen versus Affymetrix GeneChip® Citrus Genome Array), chip densities, and EST enrichment libraries used were not the same, we assume that the response detected by different experiments would be similar or even complementary and could thus help us to unravel the molecular mechanisms involved in the infection of citrus by *Ca.* Liberibacter spp.

### RT-qPCR analysis for gene expression validation

Real time PCR assays were performed with 20 genes (Additional file 6). Based on microarray analysis, 18 genes were selected for validation by RT-qPCR. Seven of them were also reported as differentially expressed in CaLas infected leaves of citrus [17]: an oxidorreductase, a kunitz family protein, miraculin-like protein, glucose-6-phosphate/phosphate transporter 2 (GPT2), a chloroplastic copper/zinc superoxide dismutase (CSD2), a beta-carbonic anhydrase (SABP3) and the transcription factor WRKY70. Two additional defense-related genes, which were not differentially expressed by our microarray results, were tested: a pathogenesis related proteins (PR1), and a phloem protein (PP2-B15), both were reported as significantly induced in citrus upon infection with CaLas [17]. The gene product specific primers were designed using Primer 3 (http://frodo.wi.mit.edu/primer3/) and IDT SciTools Real Time PCR (http://www.idtdna.com/scitools/Applications/RealTimePCR/) software tools with melting temperatures of 60°C, amplicon length of 150 to 200 bp, and a GC content of 50 to 60%. Amplicon specificity was checked by 2% (w/v) agarose gel electrophoresis and by melting-curve analysis (Additional file 6). Sequence identity was confirmed by direct sequencing of PCR products using an Applied Biosystems 3730 capillary DNA sequencer (Applied Biosystems, Foster City, CA, USA).

Relative quantification was carried out in a 96-well optical plate with an ABI PRISM 7500 FAST sequence detection system (Applied Biosystems), using the Fast SYBR green PCR master mix (Applied Biosystems). All RT-qPCR assays were performed in a 25 μL reaction using 1 × Fast SYBR green PCR master mix, 200 nM of each gene specific primer pair and 3 μL of the 1:50 diluted cDNA. The standard thermal profile was used for all amplifications. All assays were carried out in three biological replicates with three amplification replicates and a non-template control. To analyze dissociation curve profiles, the following program was run after the 40 cycles of PCR: 95°C for 15 sec, followed by a constant increase in temperature between 60 and 95°C. Raw data of fluorescence accumulation for each individual assay were imported into the R statistical package version 2.922 (R Development Core Team). Fluorescence values accumulating at each cycle were used to fit a four parameters sigmoid curve that represented each amplification curve, using the library qPCR [56]. Quantification cycles (Cq) values were then determined by the maximum of the second derivative of the fitted sigmoid curve. The efficiency of each amplification reaction was calculated by the ratio between the fluorescence value obtained in Cq and fluorescence value obtained in the amplification cycle immediately prior to Cq. The efficiency of each gene was estimated as the average of the efficiency values calculated in all amplifications of that gene. Genes used in the normalization between the different amplified samples were selected as detailed below. The comparison of means of normalized expression values among groups were performed by a nonparametric one-way ANOVA with 1000 unrestricted permutations, followed by pair-wise comparisons with Bonferroni adjustment [57]. The results were represented in graphs displaying the mean of expression levels ± mean standard error (SE) of each group relative to the control group. Two-tailed levels of significance less than or equal to 0.05 and 0.1 were considered as “significant” and “suggestive”, respectively.

### Selection of reference genes for gene expression normalization

To obtain reliable gene expression measurements, we screened candidate reference genes selected in our microarray analyses, according to the following criteria: |logFC| ≤ 0.5, average expression (AveExp ≥ 7.0), and standard deviation (stdev ≤ 0.5). Unigene transcripts were then sorted by: (1) coefficient of variation (CV), (2) standard deviation, and (3) LogFC. Based on these results, we selected TIP41-like and an importin as candidate reference genes to be tested. In addition, we decided to evaluate the expression stability of 18S ribosomal and GAPDH primers, which have been used as normalizers in previous studies [16,17,19]. Finally, we added a Polypyrimidine tract-binding protein 1 (PTB1), a SAND family protein, an Elongation factor 1-alpha (EF1α), a DIM1 homolog/YLS8 (DIM1) and a F-box family protein (FBOX) genes, which were considered as superior reference genes for normalizing gene expression in citrus in a previous systematic analysis conducted in our laboratory [58] (Additional file 7). Primer efficiencies, Cq values and normalized relative quantities (NRQ) were calculated as described above. The most stable reference genes were identified using the geNorm 3.5v (medgen.ugent.be/~jvdesomp/geNorm/) algorithm [59]. geNorm estimates an average expression stability value (*M* value) for each reference gene by the pairwise variation between a reference gene and all others reference genes tested. Stepwise exclusion of the reference genes with the lowest stability of expression (the highest *M*) allows ranking the reference genes according to their expression stability. The pairwise variation also determines the minimum number of reference genes required for a reliable normalization. In this case, we used a cut-off of 0.15, below which the inclusion of an additional reference gene is not necessary [59].

## Competing interests

The authors declare that they have no competing interests.

## Authors’ contributions

MAM and JFA planned and supervised the study. CSF conducted the biological experiments, including extraction of plant RNA. VM and MRA performed the microarray analysis. VM, PKM, and CSF contributed to the RT-qPCR validation and interpretation of the results. VM, PKM, CSF drafted the manuscript. VM wrote the final version. MAM, JFA and MRA provided intellectual input and revised the manuscript. All authors read and approved the final manuscript.

## Supplementary Material

Additional file 1 **Full list of the upregulated and downregulated citrus genes found in the microarray analyses.** Only those differentially expressed genes with a putative annotation with relation to *Arabidopsis thaliana* and with significant expression changes (adjusted p-values ≤0.05, fold change |FC| ≥ 2.0 and odds probability ≥ 0.95) are presented. Click here for file

Additional file 2 Molecular function (A) and Cellular component (B) ontologies overrepresented by Gene Set Enrichment Analysis (GSEA). Click here for file

Additional file 3 **Biological processes (BPs) overrepresented by Gene Set Enrichment Analysis (GSEA).** The up- and downregulated genes belonging to each enriched BP are displayed as TAIR codes. In some cases, more than one citrus probe ID was annotated with the same TAIR code. Details for the citrus probe ID, putative annotation and fold change of each TAIR code are listed in Additional file 4. Click here for file

Additional file 4 Differentially expressed citrus genes grouped into the biological processes enriched by Gene Set Enrichment Analysis (GSEA). Click here for file

Additional file 5 **Comparison of the expression levels of ten genes in symptomatic (SY) and asymptomatic (ASY) leaves infected with CaLas or CaLam in relation to their controls (H) by RT-qPCR.** Comparisons were performed by a nonparametric one-way ANOVA with 1000 unrestricted permutations, followed by pair-wise comparisons with Bonferroni adjustment. Levels of significance less than or equal to 0.05 and 0.1 were considered as “significant” (*) and “suggestive” (^.^), respectively. Click here for file

Additional file 6 Genes and primer sequences used for RT-qPCR assays. Click here for file

Additional file 7 Genes and primer sequences of the reference genes tested in this study. Click here for file
