# Supplementary figures and images for: Candidatus Liberibacter americanus induces significant reprogramming of the transcriptome of the susceptible citrus genotype
Source: BMC Genomics. 2013 Apr 12;14:247. doi: 10.1186/1471-2164-14-247 (PMC3635983; doi:10.1186/1471-2164-14-247)

A)

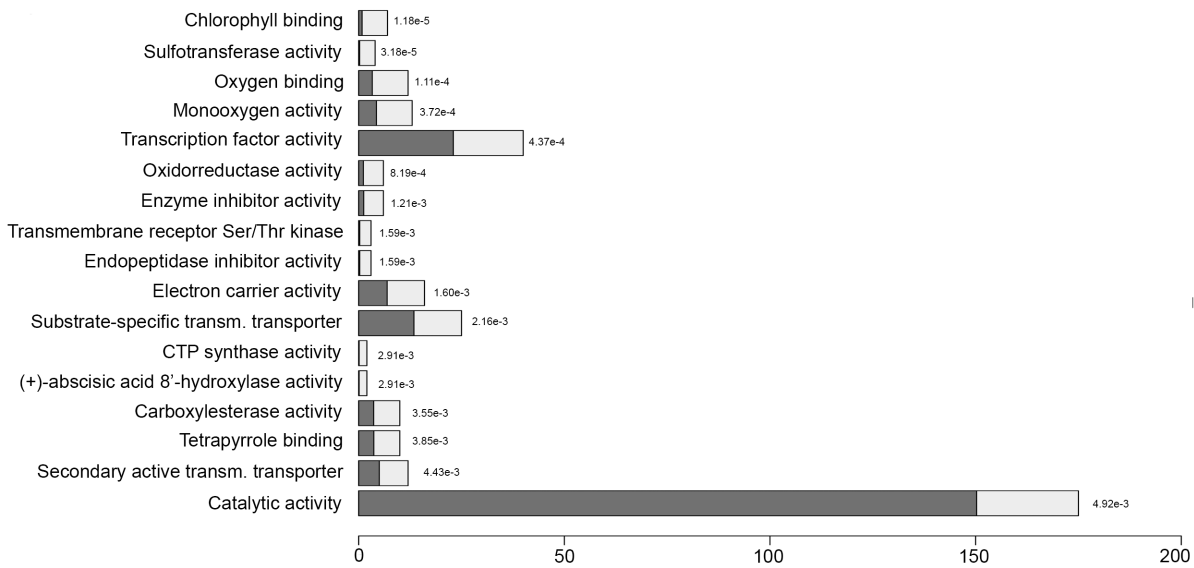

B)

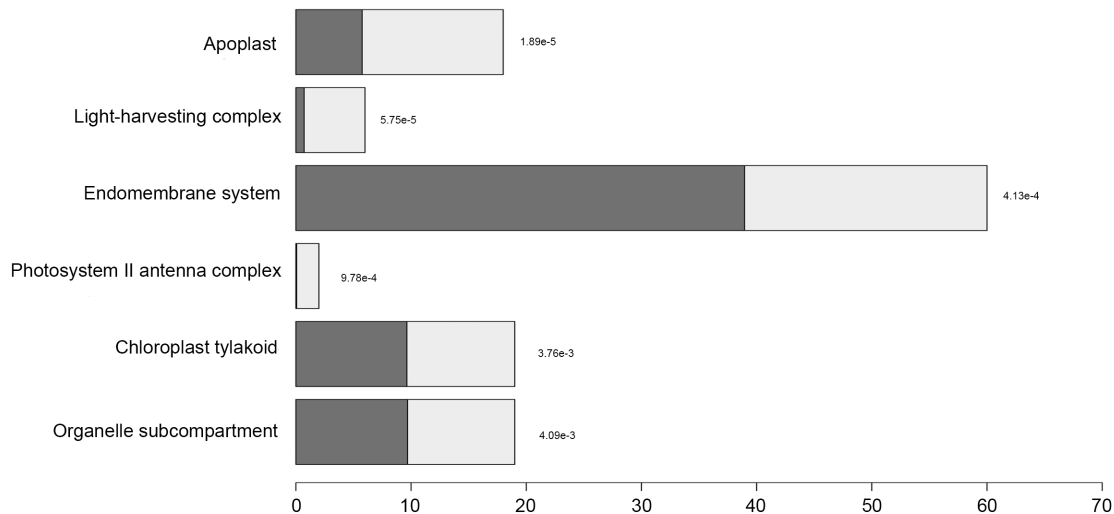

Supplement: Additional file 2 — Molecular function (A) and Cellular component (B) ontologies overrepresented by Gene Set Enrichment Analysis (GSEA). [file 1471-2164-14-247-S2.pdf]

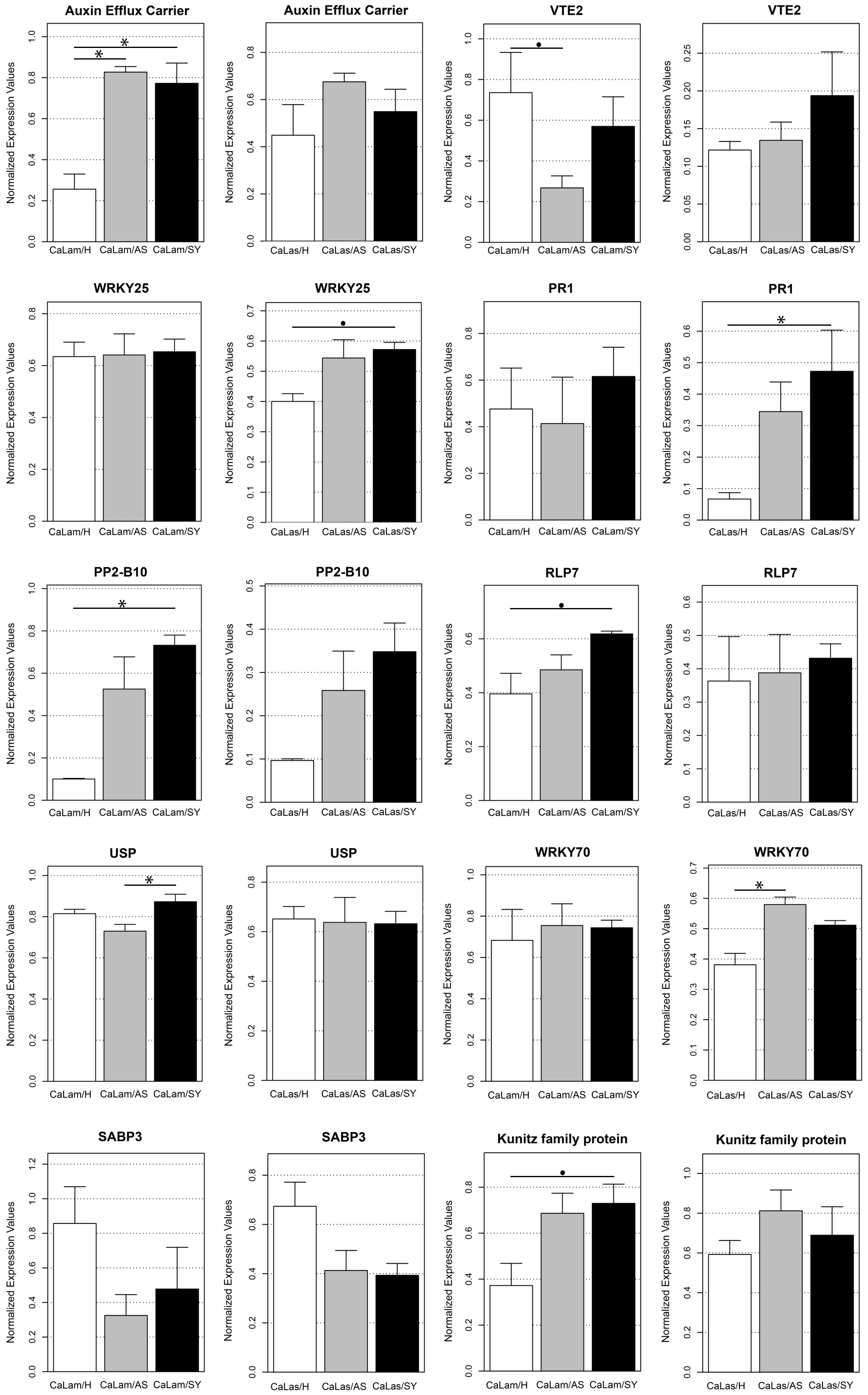

Supplement: Additional file 5 — Comparison of the expression levels of ten genes in symptomatic (SY) and asymptomatic (ASY) leaves infected with CaLas or CaLam in relation to their controls (H) by RT-qPCR. Comparisons were performed by a nonparametric one-way ANOVA with 1000 unrestricted permutations, followed by pair-wise comparisons with Bonferroni adjustment. Levels of significance less than or equal to 0.05 and 0.1 were considered as “significant” (*) and “suggestive” (.), respectively. [file 1471-2164-14-247-S5.pdf]
